# Unveiling the mandibular mystery: a case of central giant cell granuloma in posterior mandibular region—a case report

**DOI:** 10.1186/s13256-025-05613-4

**Published:** 2025-11-11

**Authors:** Aakanksha Tiwari, Suwarna Dangore-Khasbage

**Affiliations:** Department of Oral Medicine and Radiology, Datta Meghe Institute of Higher Education and Research, Sharad Pawar Dental College and Hospital, Sawangi Meghe, Wardha, Maharashtra 442001 India

**Keywords:** Central giant cell granuloma, Multinucleated, Posterior mandible, Reactive lesion, Neoplastic lesion

## Abstract

**Background:**

Previously called giant cell reparative granuloma, central giant cell granuloma is an intraosseous lesion of unclear cause that is benign but may become aggressive. It frequently crosses the midline and typically affects the anterior area of the jaw, particularly in young females. Because the posterior mandibular lesion is uncommon, especially in middle-aged people, these presentations are difficult to diagnose and have clinical significance.

**Case Presentation:**

A 37-year-old Indian female reported that she had been experiencing a painful, progressively growing bulge in her left posterior jaw for the previous 4 months. It was accompanied by paresthesia of lower left side of the face. Upon intraoral examination, a firm, nontender enlargement that stretched from the premolar to the molar region was discovered. A reactive bone lesion was suggested by a multilocular radiolucency found during radiographic assessment. Central giant cell granuloma was diagnosed after an incisional sample and histological examination. The patient had the affected side segmentally resected owing to the size and placement of the lesion. There was no recurrence observed during the follow-up period, and the postoperative recovery went smoothly.

**Conclusion:**

In contrast to the usual demographic and anatomical trends, this case illustrates a rare presentation of central giant cell granuloma in the posterior mandible of a middle-aged female. The significance of taking central giant cell granuloma into account when making a differential diagnosis for jaw swellings other than anterior region and in older age groups is highlighted by this atypical placement. By describing an uncommon site of involvement, the case contributes significantly to the limited literature on this atypical presentation. It also emphasizes the necessity of thorough clinical, radiographic, and histological correlation for precise diagnosis and treatment.

## Background

Central giant cell granuloma (CGCG), formerly known as giant cell reparative granuloma, is an intraosseous lesion of unclear etiology. While benign, it can exhibit locally aggressive behavior. It has occasionally been suggested that intraosseous bleeding and giant cells are the outcome of a reactive process that follows trauma, but pathophysiology is still unknown. According to recent data, CGCGs are thought to have an osteoclastic origin [1]. From a histological perspective, the lesions exhibit granulation tissue proliferation with multinucleated giant cells embedded in a fibrous stroma. In patients between the ages of 10 and 25 years, CGCG is responsible for about 10% of all benign tumors of the jaws that involve the mandible and maxilla. Depending on the variance in the lesion, it can show as asymptomatic, slowly growing lesions or as more severe, rapidly expanding lesions with cortical expansion, thinning, and perforations that need to be treated medically and surgically [2].

## Case presentation

A 37-year-old Indian female patient reported with chief complaint of pain and swelling in the area of her lower left back tooth region for 4 months. The swelling began modestly and increased over time to its current magnitude. The pain was explained to be severe and sporadic, that was made worse by food and momentarily eased by medications. The patient previously had paresthesia over the afflicted area and mobility of teeth in affected region. There was history of fracture of tooth in same region 2 months prior. The patient did not give any history of trauma in the affected region in recent past. Upon a general physical examination, the patient showed no notable medical history and was compliant and aware.

### Clinical findings

During extraoral examination, a tender and firm diffuse swelling measuring 4 × 6 cm in diameter was observed on the left side of the mandible. It extended antero-posteriorly from the left para-symphysis region to the mandibular angle and supero-inferiorly from the level of line joining corner of the mouth and lobule of ear up to the inferior border of the mandible.

**Fig. 1:**
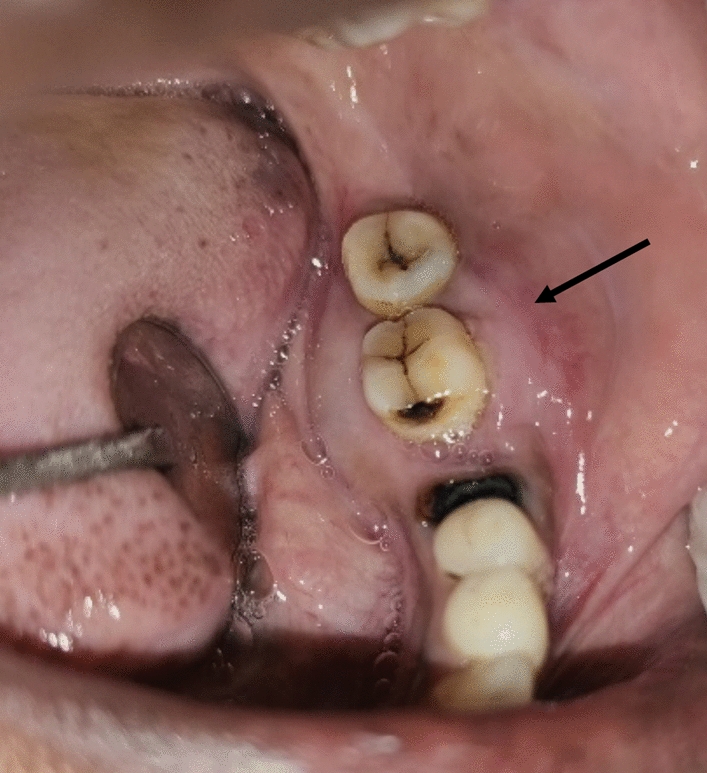
Intraoral swelling on buccal and lingual aspect in 36 to 38 region denoted by black arrow

Intraoral examination revealed diffuse intraoral swelling measuring 3 × 5 cm, extending from the 36 region to the 38 region and obliterating the buccal and lingual sulci buccolingually, the loss of distal root of 36 and mobility of 37 and 38 (Fig. 1). No crepitus or crackling was present.

### Clinical provisional diagnosis and differential diagnosis

Clinical provisional diagnosis was given as ameloblastoma while odontogenic myxoma, calcifying epithelial odontogenic tumor, odontogenic keratocyst, radicular cyst with 36 and central giant cell granuloma were well-thought-out in clinical differential diagnosis. These were listed on the basis of the location of the lesion, sex, clinical manifestation and intra-oral findings, expansion of the cortical plates, and paresthesia was noted.

### Diagnostic assessment

Radiographic assessment—orthopantomogram (OPG)—was performed as a first investigation which showed a large radiolucency in the left mandibular body with few thin, wispy septa and few loculations. The root resorption of 37, 38 and thinning of the inferior cortical border of the mandible was also noted. Computed tomography scan was performed to determine the exact location and extension of the lesion. Three-dimensional (3D) reconstruction image revealed ill-defined irregular bone destruction in left mandibular body region while axial and coronal section of CT scan revealed expansile well defined lesion in left mandibular body region. Radiographic differential diagnoses, such as radicular cyst, ameloblastoma, odontogenic myxoma and brown’s tumor, were ruled depending on the lesion borders, pattern of septation, pattern of trabeculae, and cortical expansion. Ameloblastoma was considered owing to the location of lesion in posterior mandibular body region and multilocularity of lesion, but the ill-defined borders and presence of thin wispy septa in internal structure of lesion as depicted on OPG, favors CGCG. Odontogenic myxoma also was considered due to multilocularity but the characteristic thin, straight septa, pathognomonic to the condition were not seen within the lesion. Brown’s tumor was listed pertaining to occurrence of multilocular lesion in middle aged female patient, but there was absence of any systemic illness including hyperparathyroidism.

**Fig. 2:**
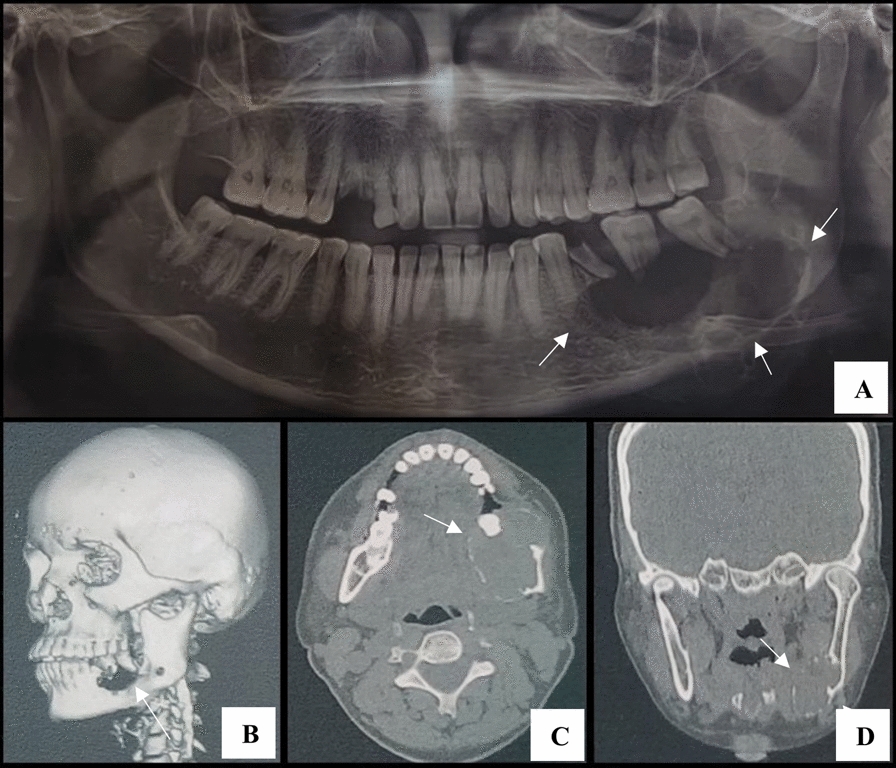
Radiographic findings: **A** Cropped orthopantomogram showing a large, multilocular radiolucency in the left mandible with resorption of the roots of teeth 37 and 38. **B** Three-dimensional computed tomography reconstruction illustrating ill-defined bone destruction. **C** Axial computed tomography view demonstrating the expansile nature of the lesion and perforation of the buccal cortical plate (arrowhead). **D** Coronal computed tomography view showing thinning of the inferior border (arrow)

Other investigations, including hematological tests to determine serum levels of calcium, phosphorus and alkaline phosphatase, were performed and values were found to be within normal limits, eventually ruling out hyperparathyroidism. The solid lesion was indicated by the negative fine needle aspiration cytology (FNAC) result (Fig. 2).

**Fig. 3:**
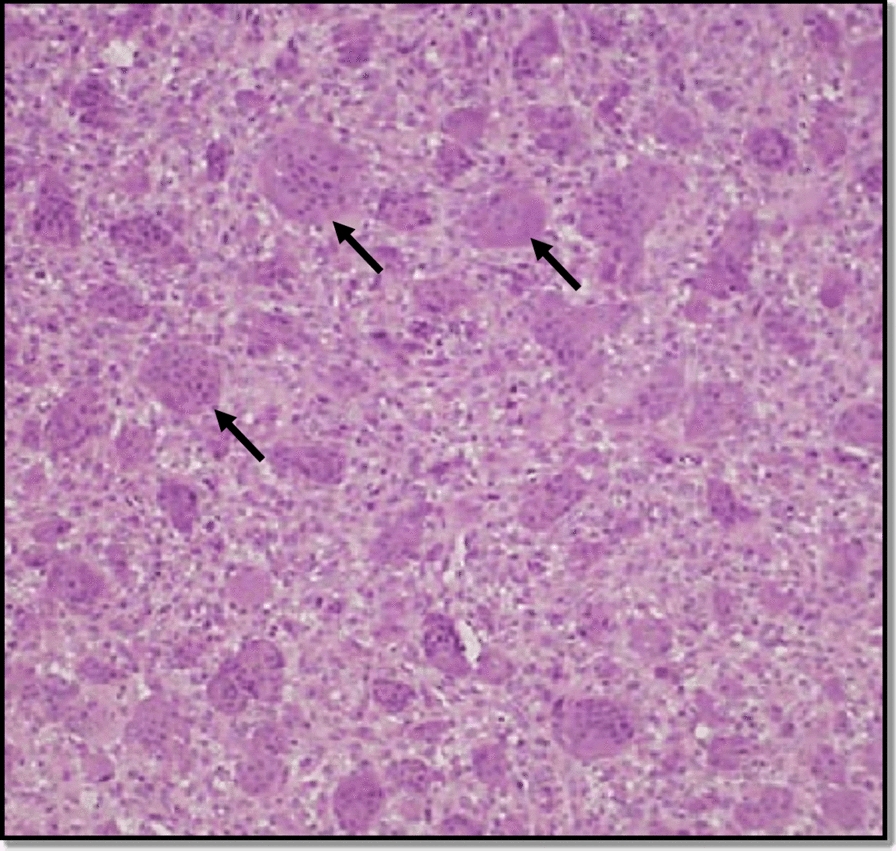
Photomicrograph with black arrows pointing multiple large multinucleated giant cells in fibrous stroma

The histological characteristics of fibro-cellular connective tissue, including fibroblasts, multiple large multinucleated giant cells, and a mild chronic inflammatory cell infiltrate primarily lymphocytes, were confirmed by incisional biopsy of the lesion (Fig. 3).

On the basis of clinical and radiological findings, clinical provisional diagnosis was given as ameloblastoma. Clinicians and radiologists were in dilemma for diagnosis of the lesion as its location and patients age suggested ameloblastoma while presence of root piece in the region of interest gave an impression of radicular cyst. Multiple investigations had to be performed to arrive at accurate diagnosis, which was finally confirmed by histopathology to be “central giant cell granuloma.” The other listed entities in differential diagnosis have been ruled out on the basis of clinical, radiological and histological findings. This marks and highlights the uniqueness of this case which posed a great challenge in diagnosis of the lesion.

### Surgical intervention

**Fig. 4:**
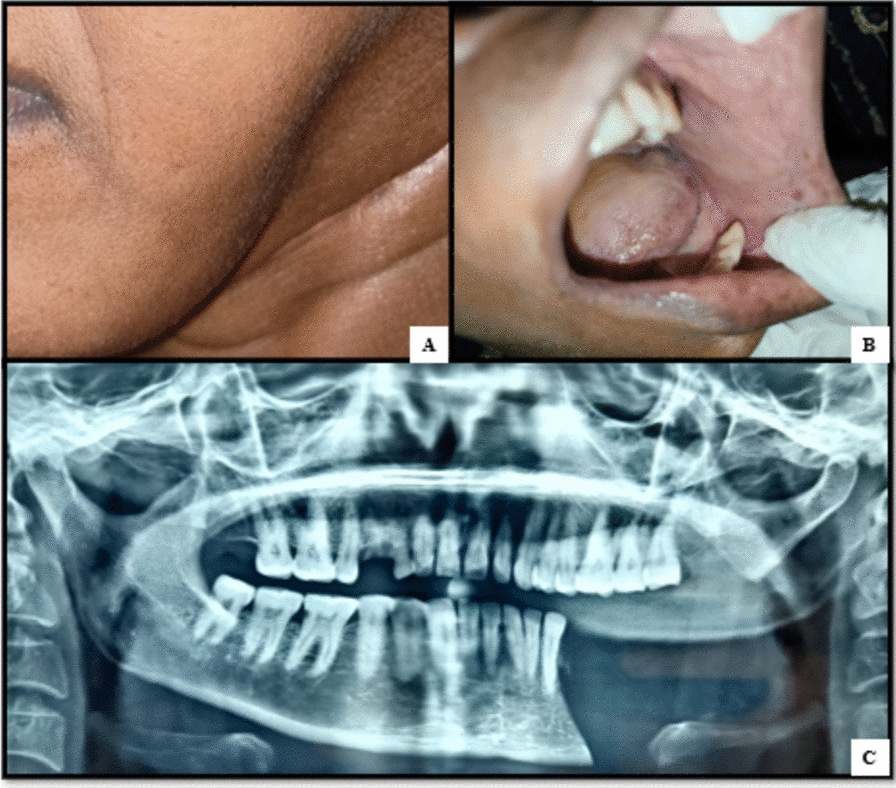
**A** Postoperative photograph showing facial disfigurement on left side of face, **B** intraoral postoperative photograph showing missing left mandibular segment distal to 34, and **C** orthopantomogram showing surgical defect involving body and ramus of mandible on left side

The involved portion of the mandible was treated with segmental mandibulectomy with safe margins. This had to be performed over other conservative management strategies owing to large lesion size, aggressive nature of the lesion, its rapid growth, and associated neurological symptoms in the form of paresthesia. The histological analysis of the removed mandible corroborated the clinical diagnosis of CGCG. After a year of follow-up, there has been no sign of a recurrence in the case as suggested by postoperative orthopantomogram (Fig. 4). Reconstruction was advised to the patient and proper counseling about the same was done prior to the surgery but even after repeated counseling patient did not give consent for the same.

## Discussion and conclusion

Jaffe originally identified CGCG as a giant-cell reparative granuloma of the mandible in 1953 [3]. A theory was proposed that the lesion is only the product of a local reparative reaction rather than a real tumor. CGCG presents as an expansile mass that is painless. The CGCG can present clinically as a slowly expanding, painless swelling or as an aggressive lesion that causes localized bone loss, root resorption, or tooth displacement [4]. Lesions are more common in women than in men, more common in the mandible than in the maxilla, and typically affect individuals under 30 years of age. The lesion is more common in the anterior region of the mandibular body and has been observed to be limited to the tooth-bearing area of the jaws [5].

Although the precise cause of CGCG is unknown, it is thought to be a non-neoplastic lesion that may have a developmental, inflammatory, or reactive origin. One possible trigger that could cause an exaggerated reparative response is trauma or intraosseous hemorrhage [6]. This can be linked with the present case as patient provided history of trauma to the tooth in the region of CGCG which lead to fracture of tooth. This could be linked as an etiologic factor for CGCG in this case. Certain incidences have been linked to hereditary disorders such neurofibromatosis type 1 and Noonan syndrome. Furthermore, because of their histological resemblances, some CGCGs may be a localized form of giant cell tumor or linked to metabolic diseases, such hyperparathyroidism, which need to be examined [6].

Giant cell granuloma’s radiologic characteristics are unclear; the lesion may show up as unilocular or multilocular radiolucency with distinct or ill-defined edges and differing degrees of cortical plate enlargement. The lesion’s radiographic appearance is not pathognomonic and can be mistaken for a number of different jaw lesions as observed in present case [7].

Treatments for CGCG of the jaws have been described using a variety of techniques. The most common therapeutic approach is curettage, either by itself or in conjunction with resection, with or without continuity loss [8]. Intralesional corticosteroid injections have been shown by some researchers to be a successful nonsurgical treatment [9]. When used as a nasal spray or subcutaneous injection, calcitonin may be useful in treating aggressive lesions [10]. The effectiveness of alpha-interferon -2a (IFN-α 2-a) injections has also been shown to vary. Depending on the anatomic location, size of lesion, clinical behavior, and involvement of the nerve or periosteum, surgical treatment is altered. However, in this instance, we had to do surgical resection and primary reconstruction using a titanium plate and autogenous bone transplant owing to cortical plate perforation [11]. In conclusion, although it is well known that the anterior region of mandible is the commonest site for CGCG. Nevertheless, it may occur in posterior region of mandible. Hence, while dealing with swelling in posterior region of mandible and interpreting a radiolucency in posterior region of mandible, along with common entities, CGCG should also be considered in the differential diagnosis. However, final diagnosis needs to be confirmed by histopathological investigation.

## Data Availability

Not applicable.

## Abbreviations

CGCG: Central giant cell granuloma
OPG: Orthopantomogram
FNAC: Fine needle aspiration cytology
INF α 2a: Interferon alpha 2a
